# Differential antimicrobial activity of two LL37 derivatives against periodontal bacteria, including enhanced efficacy against *P. gingivalis* in the presence of cigarette smoke

**DOI:** 10.3389/fmicb.2026.1804908

**Published:** 2026-04-28

**Authors:** Samaneh Keshavarz, Josefine Hirschfeld, Melissa M. Grant

**Affiliations:** 1 Periodontal Research Group, Department of Dentistry, School of Health Sciences, College of Medicine and Health, University of Birmingham and Birmingham Dental Hospital (Birmingham Community Healthcare Trust), Edgbaston, Birmingham, United Kingdom; 2 National Institute for Health and Care Research (NIHR) Birmingham Biomedical Research Center, University of Birmingham, Birmingham, United Kingdom; 3 Department of Periodontology, Operative and Preventive Dentistry, University of Bonn, Bonn, Germany

**Keywords:** antimicrobial peptides, cigarette smoke, FK13a1-NH2, KR12-NH2, LL37, periodontitis

## Abstract

**Introduction:**

Periodontitis is a plaque-induced condition that causes irreparable damage to the supporting tissues of the teeth, and tobacco smoking is a recognized exacerbating and risk factor. Antibiotic-resistant bacteria are a consequence of antibiotic overuse. Antimicrobial peptides (AMPs) are innate immune system molecules that show promise in combating antibiotic resistance.

**Methods:**

The current work investigated the efficiency of two synthetic derivatives of AMP human cathelicidin LL37, FK13a1-NH2 and KR12-NH2, in inhibiting target periodontal bacteria such as *Fusobacterium nucleatum* (*F. nucleatum*), *Porphyromonas gingivalis* (*P. gingivalis*), and *Streptococcus oralis* (*S. oralis*), in the presence of cigarette smoke extract (CSE). The amount of cigarette smoke extracted from one cigarette was considered the initial CSE maximum concentration (100%). Among the techniques used were microdilution testing, checkerboard experiments, and confocal imaging.

**Results:**

The results of the investigation showed that the two synthesized AMPs effectively inhibited the growth of *P. gingivalis* and *F. nucleatum* in the planktonic phase. Combining 500 μg/ml of KR12-NH2 with 50% CSE resulted in 98% *P. gingivalis* growth inhibition. Both peptides also significantly reduced the thickness of single-species biofilms; however, their impacts on complex, multi-species biofilms were less noticeable.

**Conclusion:**

Peptides applied in the presence of CSE were effective against *P. gingivalis*, a major periodontal pathogen. Therefore, they may be a promising alternative to standard antibiotics against periodontal bacteria regardless of smoking status, warranting further investigation.

## 1 Introduction

The human oral cavity hosts approximately 700 bacterial species that interact with each other both physically and metabolically. In healthy oral tissues, supragingival plaque is composed of non-pathogenic and aerobic species, and the composition of supragingival and subgingival plaque is similar (Sedghi et al., 2021). This balanced microbial ecosystem is maintained through host defense mechanisms, including antimicrobial peptides (AMPs), which help prevent the overgrowth of pathogenic species.

Periodontitis represents a disruption of this homeostasis and is typically initiated by plaque accumulation, where the host inflammatory response toward these biofilms plays an essential role in disease development. Characteristically, anaerobic, Gram-negative species are found in this condition, along with host inflammatory responses that cause irreversible destruction to periodontal tissues including the tooth-supporting bone (Ebersole et al., 2014).

Environmental factors including smoking can further influence both the composition of the oral microbiota and the host defense response. Tobacco use often begins during childhood or adolescence, making it a major global health concern (Grapatsas et al., 2017). Cigarette smoke contains about 400 chemicals, including unstable free radicals that cause cell damage and more stable and destructive reactive oxygen species that can stimulate the production of reactive oxygen in tissues (Higashi et al., 2014). Tobacco smoking is strongly associated with an increased risk of the incidence and progression of periodontitis (Ebersole et al., 2014). In other words, when the oral environment is exposed to cigarette smoke, the healthy microbiota turns into a pathogenic and pro-inflammatory microbiota that contributes to the development and progression of periodontal disease (Cicchinelli et al., 2023). A cumulative effect of smoking on periodontal tissues has been found indicating that individuals with greater lifetime tobacco exposure exhibit more severe periodontal disease (Leite et al., 2018). Regardless of the presence or absence of periodontal disease, smoking itself increases the abundance of *F. nucleatum* (Cheung et al., 2015). Grant et al. reported that in smokers without periodontitis, more *Streptococcus mutans* (*S. mutans*) and *Streptococcus salivarius* are seen, while in smokers with periodontal disease, *P. gingivalis* prevails (Grant et al., 2019). The inflammatory response caused by the stimulation of *P. gingivalis* is reduced in smokers, and this can be a possible explanation for the reduction of inflammatory symptoms, although the possibility of periodontitis increases in smokers (Bagaitkar et al., 2009).

Regarding the molecular mechanisms triggered by tobacco exposure, specific adhesive proteins such as FimA in *P. gingivalis* were shown to be upregulated, which could facilitate colonization and contribute to the high prevalence of periodontitis in smokers. However, the suppression of the protective bacterial capsule by cigarette smoke extract has been highlighted, which can explain the lower growth of single species biofilms in the presence of CSE in the current study (Hutcherson et al., 2015).

The three bacteria that were tested in this study were *Streptococcus oralis* (*S. oralis*), *Porphyromonas gingivalis* (*P. gingivalis*) and *Fusobacterium nucleatum* (*F. nucleatum*). *S. oralis*, a Gram-positive and non-motile bacterium, is a part of the normal oral microbiome, however, it may become an opportunistic pathogenic in infectious diseases under specific circumstances involving the breach of natural barriers, the presence of underlying health conditions, or medical interventions (Ren et al., 2021). This bacterium is an early colonizer; therefore, it plays an essential role in the formation of oral biofilms (Boisen et al., 2021). In addition, it can produce adhesins and use them to attach to host cells, and this factor also helps its colonization (Bloch et al., 2024). In Bloch et al.’s review, the authors stated the importance of understanding the pathogenicity factors of *S. oralis* and that this bacterium has diverse strains, which explains why some strains of this bacterium are much more pathogenic than others (Bloch et al., 2024). According to Gorr et al., *P. gingivalis* and *F. nucleatum* are associated with periodontal disease (Gorr, 2012). *P. gingivalis* contributes to periodontal destruction by inducing the synthesis of inflammatory cytokines, in addition, its lipopolysaccharide inhibits the differentiation of osteoblasts suppressing mineralisation and periodontal regeneration and it is believed that *P. gingivalis* proteases can degrade some antibacterial peptides (How et al., 2016). *F. nucleatum* is one of the most abundant bacteria in the oral cavity which plays an important role in the formation of dental plaque (Cheung et al., 2015).

AMPs are a critical component of the innate immune defense in the oral cavity, contributing to the regulation of microbial homeostasis at mucosal surfaces. Based on their mechanism of action, AMPs are classified into two types based on their mechanism of action: cationic peptides (e.g., cathelicidins) that destroy the bacterial membrane through electrostatic interactions (Mallapragada et al., 2017) and non-membrane binding peptides (e.g., histatins) that enter the cytoplasm and inhibit bacterial protein, nucleic acid, and cell wall synthesis, as well as enzyme activity (Salehabadi et al., 2022). AMPs are produced by salivary glands, oral epithelial cells, and neutrophils and bridge innate and acquired immunity in the body, exhibiting a wide range of antimicrobial activity against Gram-positive and Gram-negative bacteria, viruses, and fungi. In addition, these factors help maintain homeostasis between bacteria and the host’s immune system (Mallapragada et al., 2017; Ying et al., 2024).

LL37 is the only cathelicidin in humans (He et al., 2025; Khurshid et al., 2017) and is produced in the oral cavity by gingival epithelial cells and defense cells such as macrophages and neutrophils (Xia et al., 2018). Its biological effect is concentration dependent: LL37 exhibits anti-inflammatory properties in normal conditions but becomes pro-apoptotic at concentrations associated with chronic periodontal disease (Jönsson and Nilsson, 2012). Structure-function studies have shown that the middle region of LL37 is primarily responsible for antibacterial activity, whereas the N- and C-terminal regions contribute to hemolytic effects (Vallabhan et al., 2016). This has spurred the synthesis of shorter LL37-derived peptides, which have been shown to have higher antibacterial activity but lower cytotoxicity than the parent peptide (Radic and Muller, 2022). Several studies showed that smoking could reduce LL37 production, thereby aggravating periodontal disease progression (Kzar et al., 2023). Specifically, increased cotinine concentrations are associated with significantly reduced levels of LL37 in both saliva and gingival crevice fluid (Jourdain et al., 2019; Li S. et al., 2018). Yeqing et al. concluded that while LL37 is a potential biomarker and treatment option, its clinical use is limited because it is expensive, likely toxic in high doses and susceptible to proteolytic degradation; therefore, developing cheaper and more effective synthetic derivatives of LL37 is necessary in periodontal treatments (He et al., 2025). Two synthetic LL37 derivatives, FK13a1 and KR12, were utilized in this study (Madeira et al., 2022). Significant biological activity is maintained by KR12 (Jacob et al., 2013; Kamysz et al., 2020; Radic and Muller, 2022) which provides superior benefits over LL37. The superior amphipathic helicity and antimicrobial effect of KR12 alongside a drastic reduction in cytotoxicity and hemolytic activity are highlighted. Interestingly, the potential of KR12 as an anticancer agent has also been demonstrated. It has been suggested that KR12-NH2 has a more focused mechanism of action and possibly fewer side effects compared to parent peptide (Pahar et al., 2020). The inhibition of growth and biofilm formation in *Staphylococcus epidermidis* has been observed with the other derivative, FK13a1 which has shown efficacy against multidrug-resistant infections and greater selectivity for bacteria versus human cells compared to LL37 (Rajasekaran et al., 2017). The safety assessment revealed that FK13 had no sign of inducing hemolysis (Sun et al., 2024).

These shortened derivatives are viewed as promising templates because they are found to be more cost-effective to produce and are characterized by a lower number of protease cleavage sites than the original 37-residue sequence (Voronko et al., 2025). Because these peptides are significantly shorter than LL37, their synthesis is easier and cost-effective (Ridyard and Overhage, 2021).

**FIGURE 1:**
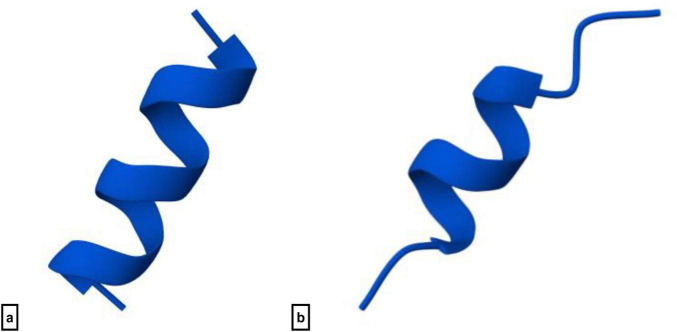
The secondary structure of FK13a1 **(a)** and KR12 **(b)**. AlphaFold 3 was used to model the FK13a1 peptide, and the result was a top-ranked structure with a global pLDDT of 93.66, showing high confidence in the secondary structure prediction. Nevertheless, the model also displayed a low pTM of 0.04 and a percent disordered value of 1.0. These measurements highlight a short, isolated peptide’s remarkable inherent flexibility over a fixed, rigid fold. L-amino acids (tryptophan, lysine, arginine, isoleucine, valine, and leucine) make up the sequence, which produces a highly cationic molecule. The KR12 model was characterized by a global pLDDT of 90.82, indicating high confidence in the secondary structure. The model reported a fraction disordered value of 1.0 and a low pTM of 0.03, indicating that the peptide is highly flexible as well and likely lacks a single, rigid tertiary fold in isolation. The peptide is composed entirely of L-amino acids, including arginine, lysine, phenylalanine, aspartic acid, and leucine, which contribute to its specific amphipathic and electrostatic properties.

The amino acid sequence of FK13a1 (WKRIVRRIKRWLR) was derived from LL37 amino acids 17–29 sequence (FKRIVQRIKDFLR) by specific amino acid substitutions, phenylalanine (F) to tryptophan (W) at position 1, glutamine (Q) to arginine (R) at position 6, aspartic acid (D) to arginine (R) at position 10, and phenylalanine (F) to tryptophan (W) at position 11. Tryptophan is known for its ability to anchor into microbial membranes (Mishra et al., 2018), while Arginine provides a strong positive charge (Yang et al., 2018). The antimicrobial peptide prediction website CAMPR4 indicates that FK13a1-NH2 has a high antibacterial activity probability (1.00). Its net positive charge of +7 allows it to interact with negatively charged bacterial membranes. The AlphaFold 3 web server, which predicts peptide secondary structures, indicates that FK13a1 primarily forms an α-helix in its central region (residues IVRRIKR), with unstructured coiled regions at its N-terminus (WKR) and C-terminus (WLR). This prediction is supported by high helix probabilities and low coil/sheet probabilities in the central region (Figure 1a). For FK13a1, the Kyte-Doolittle GRAVY index is around −0.411, suggesting that the peptide is hydrophilic (Table 1).

**TABLE 1:** Physicochemical and predictive descriptors of FK13a1 and KR12.

AMP	FK13a1 (WR-13)	KR12
Sequence	WKRIVRRIKRWLR	KRIVQRIKDFLR
Length	13 Amino Acids	12 Amino Acids
Net charge	+7 (Highly Cationic)	+5 (Cationic)
Hydrophobicity (GRAVY)	−0.411	−0.667
Secondary structure	Central α-helix (IVRRIKR)	Potential α-helix
Antimicrobial activity prediction (CAMPR4)	1.00	∼0.95+

The amino acid sequence of KR12 is KRIVQRIKDFLR (residues 18-29 of LL37). This derivative was obtained by truncating LL37 and adding an amide group to the C-terminal end. Its net positive charge of +5 enhances the interaction with negatively charged bacterial membranes and enhances antibacterial activity. A high probability of antibacterial activity (0.99) is predicted by the CAMPR4 server and AlphaFold 3 predictions suggest this peptide contains an alpha-helical core (residues VQRIKDF) as well as coiled N-terminal (KRI) and C-terminal (LR) sections (Figure 1b). The Kyte-Doolittle GRAVY index of −0.667 suggests that KR12-NH2 is primarily hydrophilic (Table 1).

Because these two peptides are both derived from LL37, their primary targets fall into two categories including bacterial components for killing infection and human signaling pathways for healing (Sun et al., 2024). Li et al. identified that KR12 targets human bone marrow stem cells to help them turn into bone cells. It does this by activating the BMP2 gene and the BMP/SMAD signaling pathway. This makes KR12 a potential target for treating bone infections (osteomyelitis) because it kills the bacteria and helps the bone regrow (Li S. et al., 2018). Like most antimicrobial peptides, KR12 doesn’t always have a single lock-and-key protein target. Instead, it targets the bacterial cell membrane (Lei et al., 2024). Research shows it binds strongly to LPS (endotoxins) on the surface of Gram-negative bacteria. This targets the infection and prevents the toxic immune overreaction (sepsis) that LPS usually causes (van Zyl and Coburn, 2024).

FK13 targets the outer membrane of tough bacteria like *Pseudomonas aeruginosa*. It acts as a helper, punching holes in the membrane so that other antibiotics (like Vancomycin) can get inside. It also disrupts the proton motive force essentially short-circuiting the battery that powers the bacterial cell.

Recent research focuses on using FK13 in hydrogels to target biofilms. By breaking down the biofilm, it targets the infection at its most resistant stage (Wang et al., 2025).

The indiscriminate use of antibiotics has led to increased resistance, making the treatment of bacterial infections challenging. In the dental field, antibiotics are also prescribed irregularly, contributing to this global challenge and complicating the results of dental and periodontal treatments. Therefore, minimizing the prescription of antibiotics is essential, and AMPs are potential alternatives (Froum and Weinberg, 2015; Rima et al., 2021; Xuan et al., 2023). The unique mechanism of action of AMPs against bacteria is physically destroying the microbial cell membrane by creating transmembrane channels or pores result in rapid death. It is hard for a bacterium to redesign its entire membrane structure to prevent this physical attack; therefore, AMPs are less likely to cause antibiotic resistance, and this makes them a potentially promising option in periodontal treatments (Liu et al., 2025; Luong et al., 2020; Montoya et al., 2023).

The purpose of this study was to explore the efficacy of FK13a1-NH2 and KR12-NH2 in suppressing the growth of periodontal pathogens in the presence and absence of cigarette smoke, to establish whether they might be utilized in periodontal patients in the future, independent of smoking status.

## 2 Materials and methods

### 2.1 Bacterial strains and culture

Bacterial strains used in this study, including *P. gingivalis* (ATCC 33277), *S. oralis* (CCUG 24891), and *F. nucleatum* (ATCC 23726) were originally provided by the ADA Forsyth Institute (Cambridge, MA, United States). *P. gingivalis* was grown on blood agar (ThermoFisher Scientific, Waltham, MA, United States), whilst *F. nucleatum* and *S. oralis* were grown on Schaedler anaerobic agar (Sigma-Aldrich, St. Louis, MO, United States). All bacteria were grown at 37°C in an anaerobic atmosphere (80% N2, 10% CO_2_, 10% H2). The planktonic bacteria were grown for 15–18 h, and biofilms were grown for 72 h in brain heart infusion (BHI) broth (Thermo Scientific, Oxoid, Basingstoke, United Kingdom). To promote bacterial growth, Hemin (Sigma-Aldrich H9039) and Vitamin K1 (Sigma-Aldrich V3501) were added to the BHI broth. For the experiments in this study, a concentration of 5 μg/ml of hemin and 1 μg/ml of vitamin K1 was desired, according to CLSI. Metronidazole, an effective antibiotic against anaerobic bacteria (Pankuch et al., 1993) routinely used in dentistry, was selected as a positive control agent in the *F. nucleatum* experiments (Adha et al., 2017; Bullman et al., 2017). The use of azithromycin as an effective antibiotic against *P. gingivalis* for positive control in the experiments was determined based on previous studies (Lo Bue et al., 1997). EUCAST provides breakpoints for the streptococci group, which includes *S. oralis*, for penicillin and other beta-lactams such as amoxicillin. Thus, amoxicillin was selected as the positive control in the *S. oralis* experiments (Larsson Wexell et al., 2016). Growth curves for the species used were established before completing these experiments and demonstrated that the organisms were in the logarithmic phase.

### 2.2 Cigarette smoke preparation

A modified version of the instrument used in Agraval et al. (2022) was employed in this investigation to extract cigarette smoke. Cigarettes [Marlboro Red as a popular brand (Pauwels et al., 2020)] were stored in their packaging in a dry and cool place according to the manufacturer’s recommendation. According to Supplementary Figure 1, a modified 200 ml syringe was prepared for this purpose. The smoke extraction process was done under the fume hood cupboard. The method of extracting cigarette smoke in this study was non-continuous, or puff based. This method simulates smoking by smokers. For the smoke extraction, three puffs of 18 s were done at a speed of 10 ml/s. After the first puff, the syringe was separated from the rubber and its cap was placed immediately; then, the syringe was shaken for 30 s to mix the cigarette smoke with PBS completely. In a 200 ml syringe containing 20 mL of PBS, the puff volume was 180 ml. The puff steps were continued until the cigarette burned completely up to the beginning of the filter. For the last time, the syringe was shaken for 30 s so that the cigarette smoke was completely mixed with PBS. The mixture of smoke and PBS was filtered through a sterile 0.22 μm syringe filter. PH was maintained at 7.4. Cigarette smoke stocks were prepared and stored at –20°C. This amount of extracted cigarette smoke was considered the CSE maximum concentration (100%) and was diluted in BHI for the experiments.

### 2.3 Peptide preparation

The lyophilized AMPs, FK13a1-NH2, and KR12-NH2, synthesized (Jacob et al., 2013; Rajasekaran et al., 2017) and purified to 91% by ProteoGenix (Schiltigheim, France); were dissolved in sterile distilled water and stored in aliquots at −20°C.

### 2.4 Minimum inhibitory concentration method (microdilution method)

Antimicrobial peptide stocks were made by combining 10 mg of the peptide powder with 1 mL of sterile distilled water in a universal tube and vortexing the solution.

Each well of a 96-well microplate was inoculated with 100 μl of AMP and 100 μl of a log-phase bacterial suspension, adjusted to an optical density (OD600) of 0.01. The microplate was incubated 24 h before the OD600 measurements were determined using a universal microplate reader (Lutgring et al., 2023).

### 2.5 Growth of planktonic bacteria in the presence of cigarette smoke

Bacterial overnights were prepared in BHI broth in an anaerobic setting. To achieve the desired OD600, the overnight culture was diluted in fresh BHI broth. CSE was serially diluted twofold in BHI. Bacteria from BHI served as growth controls. After 24 h of incubation, optical densities were measured at 600 nm using a Universal Microplate Reader (Elx 800, BIO-TEK), and data were collected. Each experiment involved three biological replicates.

### 2.6 Treatment of the planktonic bacteria with CSE and AMP (checkerboard assay)

The checkerboard protocol is a standard method used to evaluate the combined effects of cigarette smoke and antimicrobial agents (Nelson and Rosowsky, 2001). Different concentrations of the antimicrobial peptide were added to the column of a microtiter plate. Different concentrations of cigarette smoke extract were added to the rows. The test microorganism was added to each well. Each well in a 96-well plate received 100 μl of bacteria suspension, 50 μl of 2 × CSE, and 50 μl of AMP, and a two-fold serial dilution to achieve different concentrations of CSE and AMP. The final maximum concentration of CSE was 50%. The highest concentration of AMP was 500 μg/ml. The plate was incubated, and the microbial growth was assessed after 24 h using the Microplate Alamar Blue assay (MABA), as described in section 2.7.

### 2.7 MABA

Due to the opacity of FK13a1-NH2 solutions, especially when mixed with CSE, Alamar blue assay was utilized in the planktonic checkerboard experiments (Rampersad, 2012; Yajko et al., 1995). The filter sterilized Alamar Blue stock solution was at 1 mg/ml concentration in PBS of which 20 μl (10% (v/v) of the medium volume) was added to each well that already contained 200 μl AMP/CSE/AS/bacteria and plates were re-incubated at 37°C. Wells were observed every 10 min for a color change of negative control from blue to pink. Fluorescence was measured in the Tecan plate reader (infinite 200Pro, Serial number: 1205004470) in top-reading mode with excitation at 535 nm and emission at 590 nm. Absorbance reading was at 570 nm wavelength (McBride et al., 2005).

### 2.8 Single and multi-species biofilm set-up

A few colonies of bacteria were inoculated into 5 ml of BHI broth and incubated overnight in an anaerobic chamber. The overnight bacterial culture was centrifuged (Hettich Zentrifugen, Germany) at 3,000 rpm for 10 min. The supernatant was removed, and the bacterial pellet was washed by resuspending it in 5 ml of PBS, followed by centrifugation. The final pellet was resuspended in 5 ml of BHI using a vortex for 1 min. For mono-species biofilms, each type of bacteria was adjusted to an initial OD600 of 0.1 in BHI and added to wells of a sterile 96-well microtiter plate. The total volume of bacterial suspension in each well was kept at half of the total well volume. For multi-species biofilms, a mixed bacterial suspension was created by combining equal volumes of each targeted bacterial species, each adjusted to an initial OD600 of 0.1 in BHI. This mixed inoculum was then added to the wells on day zero. All well plates were incubated at 37°C in an anaerobic chamber for 72 h. The supernatant was removed and replaced with fresh media, along with any experimental treatments (CSE and/or AMPs), every 24 h until 72 h (Müsken et al., 2010).

### 2.9 Biofilm LIVE/DEAD staining protocol

Biofilms grown in 96-well black imaging microplates (Imaging microplate, Cat. No. 4TI-0223, Azenta Life Sciences, Abingdon, United Kingdom) for 72 h were prepared for staining as described here. The supernatant was carefully removed from each well without dislodging the biofilm. In a Falcon tube covered with foil, 3 μl of SYTO 9 dye and 3 μl of propidium iodide dye from a Filmtracer™ LIVE/DEAD Biofilm Viability Kit (ThermoFisher Scientific, Waltham, MA, United States), and 1 mL of phosphate-buffered saline (PBS) were mixed to create a fluorescent staining solution. Ten biofilms could be stained with this amount. 50 μl of 4% paraformaldehyde was added to each well, and after 10 min of fixation, the samples were washed with PBS. Each biofilm sample was then stained with 100 μl of the prepared fluorescent staining solution. Then, the microplate was wrapped in foil and left in a hood for 30 min in the dark. To remove any remaining dye, the wells were washed with PBS after incubation (Shailaja et al., 2022). Finally, 50 μl of PBS was added to each well to keep the biofilms from drying out.

### 2.10 Confocal laser scanning microscopy

In the dark, samples were imaged with Confocal Laser Scanning Microscopy (CLSM 700, Zeiss, Germany) using a 20x objective at 488 nm/<550 nm for SYTO^®^ 9 and 555 nm/ > 550 nm for propidium iodide (Mountcastle et al., 2021). The maximum thickness of the biofilms was estimated by obtaining z-stack horizontal images at 1.3 μm intervals. Biofilms were grown in triplicate in 96-well vision plates (Imaging microplate, Cat. No. 4TI-0223, Azenta Life Sciences, Abingdon, United Kingdom) for each experiment, and images were acquired using Zeiss Zen 2011 software.

### 2.11 Quantification of red/green areas in confocal images

A modified version of the method presented in the study by Mountcastle et al. was used to calculate the amount of red and green areas in confocal images. For this purpose, raw Z-stack images were prepared using ImageJ software. Briefly, the color channels were separated for each image slide. The distribution of pixel intensities was visible in the histogram in the threshold dialogue box. Parts of the image that corresponded to foreground pixels were highlighted in color. The same threshold settings were selected for all images. The “Dark background” option was left unselected because the stained cells in the images were lighter than the background. It was ensured that the highlights represented cells, not background or other non-cellular debris. After closing the threshold dialogue box, the “Particle Analysis” command was run for each slide, and the size parameter was selected to improve particle detection (Mountcastle et al., 2021).

### 2.12 Statistical analysis

Statistical analysis was performed using GraphPad Prism LLC 10.6.1 (799), Boston, Massachusetts. Data were analysed with Student’s *t*-tests, One-way or Two-way ANOVA after determining normal distribution by Shapiro-Wilk test. The confocal pictures were analysed using Dunnett’s multiple comparison test. *P* < 0.05 were considered as statistically significant.

## 3 Results

### 3.1 Dose-dependent and combined antimicrobial activity of FK13a1-NH2, KR12-NH2, in the presence CSE, against planktonic bacteria

**FIGURE 2:**
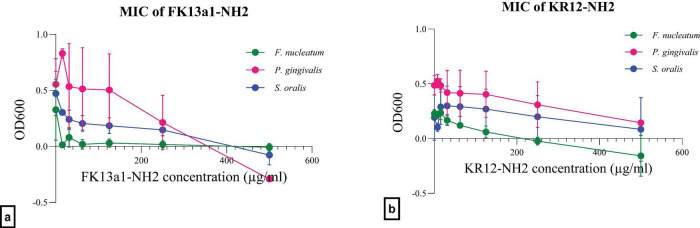
The efficacy of the AMPs against targeted planktonic bacteria using MIC assay. The value points and error bars represent mean and SD, respectively. The experiment involved three biological replicates. **(a)** FK13a1-NH2. **(b)** KR12-NH2.

The effectiveness of FK13a1-NH2 and KR12-NH2 against planktonic *F. nucleatum*, *P. gingivalis*, and *S. oralis* was assessed using the microdilution technique. The MIC of FK13a1-NH2 for *F. nucleatum* was 15.62 μg/ml, which was defined as the lowest peptide concentration with no visible growth (OD600 test/control < 0.1) (Jiang et al., 2018). The inhibition of *P. gingivalis* and *S. oralis* with 500 μg/ml of FK13a1-NH2 was 100%. The MIC of KR12-NH2 for *F. nucleatum* was 250 μg/ml and the inhibition of *P. gingivalis* with 500 μg/ml of KR12-NH2 was 70.24 ± 6.19%. This peptide did not show inhibiting effects on *S. oralis*. The data can be seen in Figure 2 and Table 2.

**TABLE 2:** The summary of single-agent and combination effects of FK13a1-NH2, KR12-NH2, and CSE on *F. nucleatum*, *P. gingivalis*, and *S. oralis* in their planktonic phase.

Condition	Organism	Concentration(s)	Primary result	% Inhibition/effect	Figure reference(s)
FK13a1-NH2	*F. nucleatum*	15.62 μg/mL	Inhibition	> 90%	Figure 2a
	*P. gingivalis*	500 μg/mL	Inhibition	100%	
	*S. oralis*			100%	
KR12-NH2	*F. nucleatum*	250 μg/mL	Inhibition	> 90%	Figure 2b
	*P. gingivalis*	500 μg/mL	Inhibition No Effect	70.24 ± 6.19% 0%	
	*S. oralis*				
CSE	*F. nucleatum*	50%	Inhibition	58.7 ± 11%	Figure 3
	*P. gingivalis*			59.3 ± 15.40%	
	*S. oralis*			67.3 ± 5%	
FK13a1-NH2 + CSE	*F. nucleatum*	500 μg/mL AMP + 50% CSE	Antagonistic	85.82 ± 22%	Figure 4a
	*P. gingivalis*		Effect	86.6 ± 23.2%	Figure 4b
	*S. oralis*		Growth Promotion	Increased Growth	Figure 4c
KR12-NH2 + CSE	*F. nucleatum*		Antagonistic Effect	85.82 ± 21.7%	Figure 5a
	*P. gingivalis*		Synergistic Effect	98.37 ± 1.76%	Figure 5b
	*S. oralis*		Growth Promotion	Increased Growth	Figure 5c

The variation may be related to the inherent structural complexity of the biofilm matrix, which can occasionally lead to uneven peptide penetration or localized survival of bacterial clusters.

**FIGURE 3:**
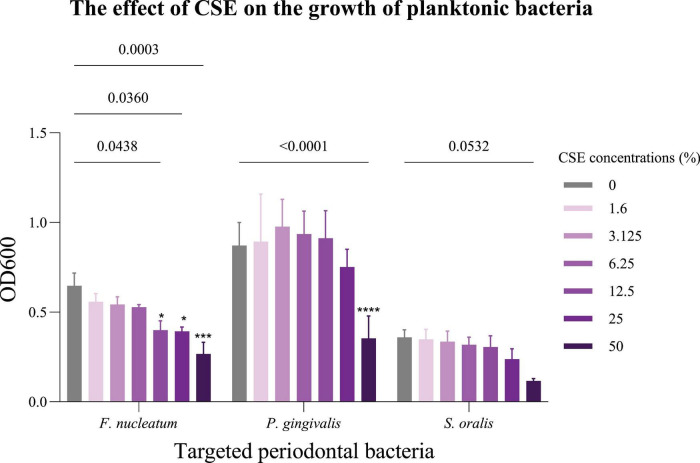
The effect of CSE on bacteria. A Two-way ANOVA was done. Mean values and standard deviations are shown. *p < 0.05, ***p < 0.001, ****p < 0.0001 compared to 0% CSE, *n* = 3.

The effect of different concentrations of CSE on the growth of *F. nucleatum*, *P. gingivalis*, and *S. oralis* was assessed. The smoke from one cigarette in PBS was set as the maximum CSE concentration of 100%. It was used in a two-fold dilution series, with 1.6% CSE as the minimum concentration in BHI. The negative control was not exposed to CSE. 50% CSE reduced the growth of planktonic *F. nucleatum*, *P. gingivalis*, and *S. oralis* by 58.7 ± 11% (*p* < 0.001), 59.3 ± 15.40% (*p* < 0.001) and 67.3 ± 5% (*p* = 0.05), respectively (Figure 3 and Table 2).

**FIGURE 4:**
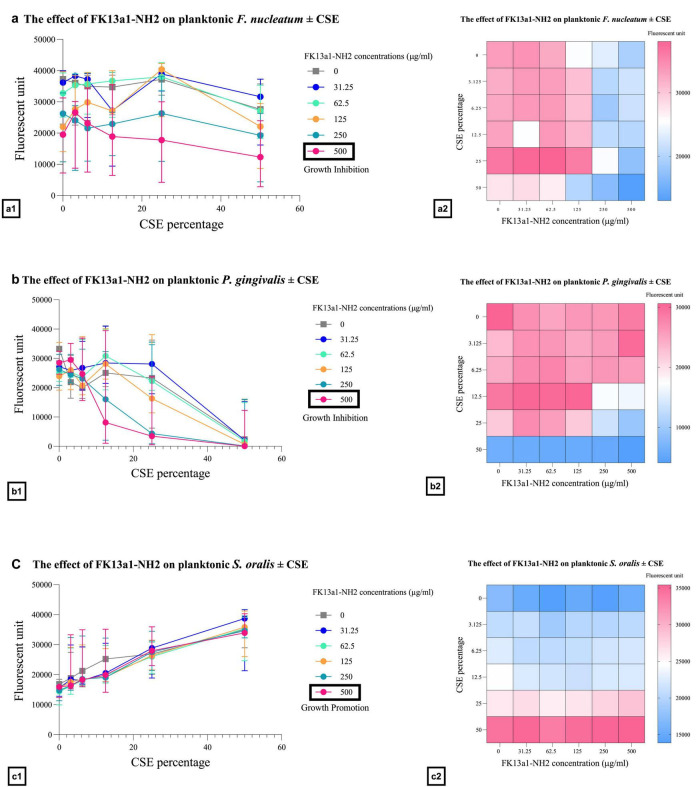
The efficacy of FK13a1-NH2 on planktonic bacteria in the presence of CSE. The line graphs and heat map show the treatment of the bacteria with FK13a1-NH2 and CSE. The value points and error bars represent the mean and SD, respectively. The experiment involved three biological replicates. **(a)** *F. nucleatum*. **(b)** *P. gingivalis*. **(c)** *S. oralis*.

**FIGURE 5:**
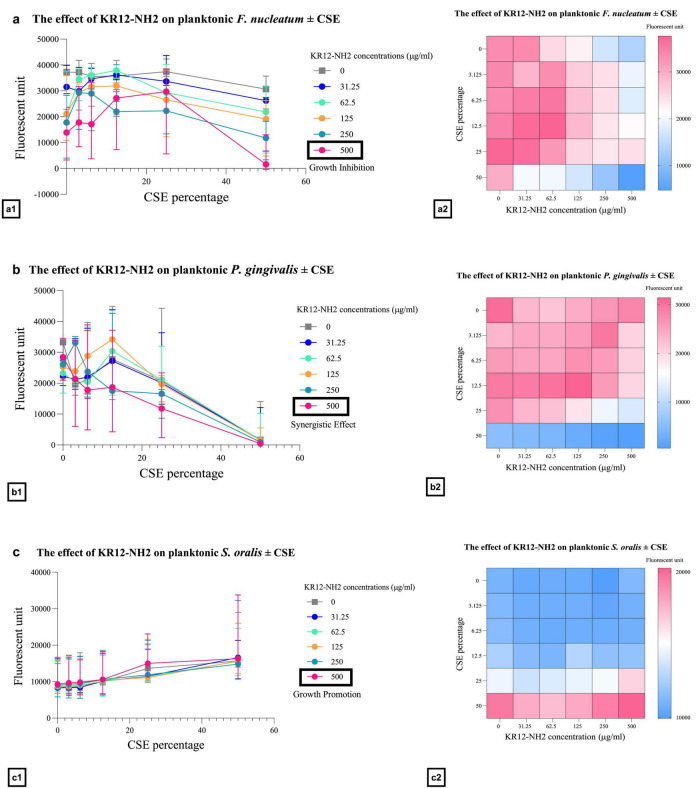
The efficacy of KR12-NH2 on planktonic bacteria in the presence of CSE. The line graphs and heat map show the treatment of the bacteria with KR12-NH2 and CSE. The value points and error bars represent the mean and SD, respectively. The experiment involved three biological replicates. **(a)** *F. nucleatum*. **(b)** *P. gingivalis*. **(c)** *S. oralis*.

The combined effects of FK13a1-NH2 and CSE on *F. nucleatum*, *P. gingivalis*, and *S. oralis* were evaluated using the checkerboard assay. The treatment of *F. nucleatum* with the combination of 500 μg/ml FK13a1-NH2 and 50% CSE resulted in 85.82 ± 22% inhibition. Combining 500 μg/ml peptide with 50% CSE resulted in 86.6 ± 23.2% *P. gingivalis* inhibition. Conversely, treating *S. oralis* with the same combination resulted in increased growth. The combined effects of KR12-NH2 and CSE on *F. nucleatum*, *P. gingivalis*, and *S. oralis* were as follows: peptide at 500 μg/ml and 50% CSE combined led to inhibition of *F. nucleatum* growth of 86%. The combination of peptide and 50% CSE resulted in a 98% inhibition of *P. gingivalis* growth. Again, when KR12-NH2 was applied to *S. oralis* at 500 μg/ml in the presence of 50% CSE, increased growth was observed. The data are shown in Figures 4, 5 and Table 2.

### 3.2 Reduction of biofilm thickness and inhibition of formation by peptides and cigarette smoke under single- and multi-species conditions

**FIGURE 6:**
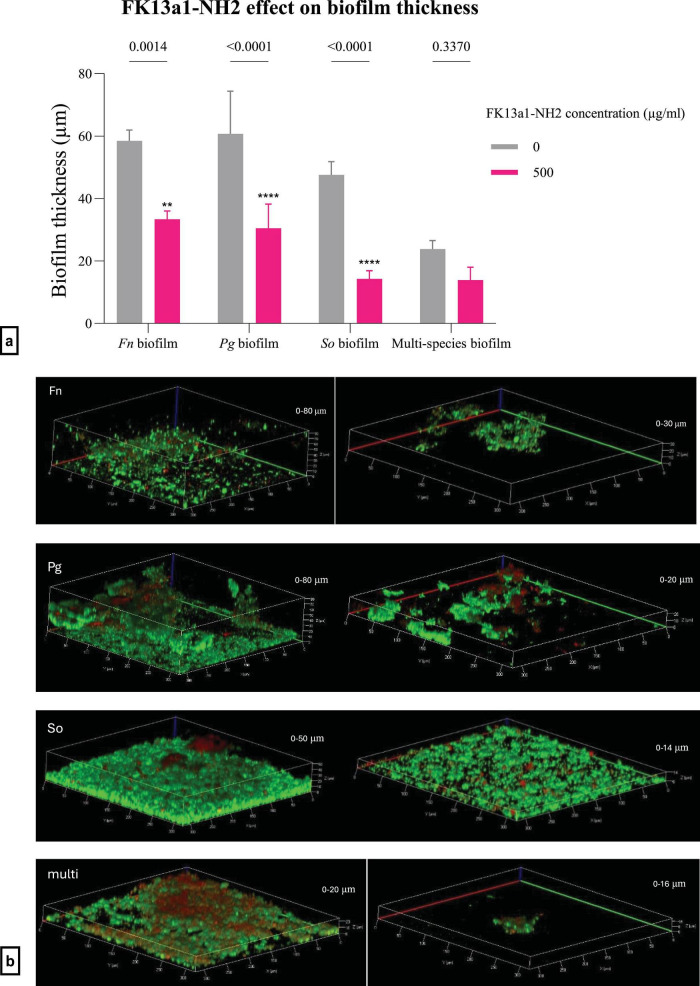
**(a)** The efficacy of FK13a1-NH2 on biofilm thickness using confocal imaging and live/dead staining. Statistical analysis was by two-way ANOVA. The value points and error bars represent the mean and SD, respectively. The experiment involved three biological replicates. The comparison within each group is with no FK13a1-NH2 (*Fn*, *Fusobacterium nucleatum*; *Pg*, *Porphyromonas gingivalis*; *So*, *Streptococcus oralis*). **(b)** The biofilm thickness measurements and live/dead staining using confocal imaging. The images are related to *F. nucleatum*, *P. gingivalis*, *S. oralis* and multi-species biofilms, respectively, from top to bottom. In each row, the left-hand side image is the control, and the right-hand side image is the treatment with 500 μg/ml FK13a1-NH2. The interval in the z-stack was 1.30 μm (*Fn*, *Fusobacterium nucleatum*; *Pg*, *Porphyromonas gingivalis*; *So*, *Streptococcus oralis*). The magnification was 20x. **p < 0.01, ****p < 0.0001.

**FIGURE 7:**
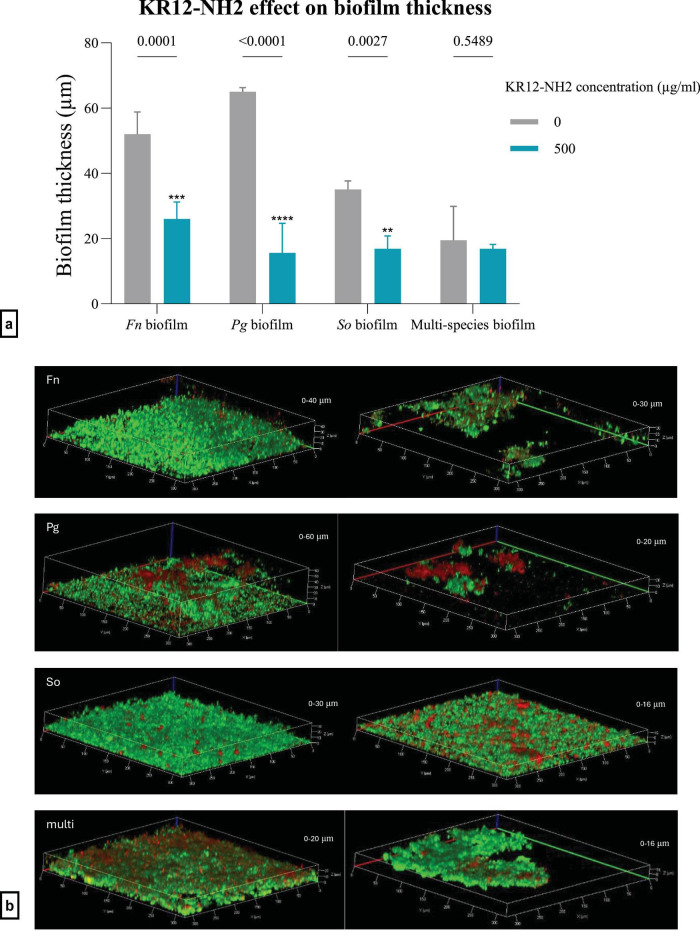
**(a)** The efficacy of KR12-NH2 on biofilm thickness using confocal imaging and live/dead staining. Statistical analysis was by two-way ANOVA. The value points and error bars represent the mean and SD, respectively. The experiment involved three biological replicates. The comparison within each group is with its own negative control (*Fn*, *Fusobacterium nucleatum*; *Pg*, *Porphyromonas gingivalis*; *So*, *Streptococcus oralis*). **(b)** The biofilm thickness measurements and live/dead staining using confocal imaging. The images are related to *F. nucleatum*, *P. gingivalis*, *S. oralis* and multi-species biofilms, respectively, from top to bottom. In each row, the left-hand side image is the control, and the right-hand side image is the treatment with 500 μg/ml KR12-NH2. The interval in the z-stack was 1.30 μm (*Fn*, *Fusobacterium nucleatum*; *Pg*, *Porphyromonas gingivalis*; *So*, *Streptococcus oralis*). The magnification was 20x. **p < 0.01, ****p < 0.0001.

Biofilms of single and multiple species were assessed microscopically with FK13a1-NH2 exposure (Figure 6a). The thickness of single-species biofilms was significantly decreased by FK13a1-NH2 exposure. The *p*-values for treated vs. untreated *F. nucleatum*, *P. gingivalis* and *S. oralis* biofilms were < 0.001 for each. There was no significant decrease in the thickness of the multi-species biofilms, with a *p*-value of 0.34 (Figure 6b). KR12-NH2-exposed single-species biofilms had significantly reduced thickness for *F. nucleatum*, *P. gingivalis* and *S. oralis* (*p* = 0.0018, 0.0003, and 0.0418, respectively). However, there was no evident change in the biofilm formation of multi-species biofilms (*p* = 0.0946) (Figure 7a). The confocal images are shown in Figure 7b.

**FIGURE 8:**
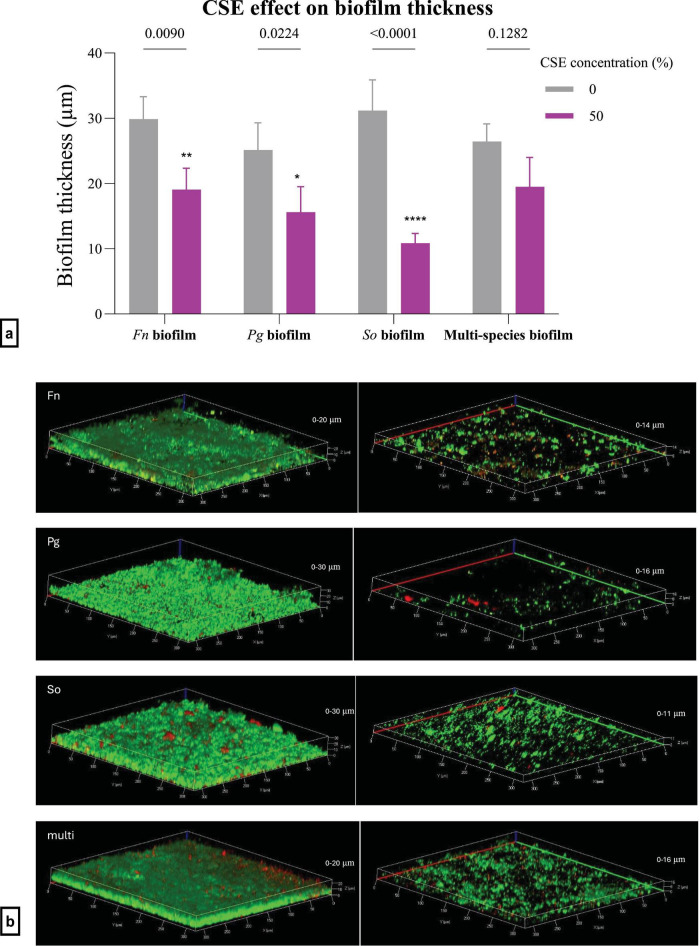
**(a)** The efficacy of 50% CSE on biofilm thickness using confocal imaging and live/dead staining. Statistical analysis was by two-way ANOVA. The value points and error bars represent the mean and SD, respectively. The experiment involved three biological replicates. The comparison within each group is with its own negative control (*Fn*, *Fusobacterium nucleatum*; *Pg*, *Porphyromonas gingivalis*; *So*, *Streptococcus oralis*.). **(b)** The biofilm thickness measurements and live/dead staining using confocal imaging. The images are related to *F. nucleatum*, *P. gingivalis*, *S. oralis* and multi-species biofilms, respectively, from top to bottom. In each row, the left-hand side image is the control, and the right-hand side image is the treatment with 50% CSE. The interval in the z-stack was 1.30 μm (*Fn*, *Fusobacterium nucleatum*; *Pg*, *Porphyromonas gingivalis*; *So*, *Streptococcus oralis*). The magnification was 20x. *p < 0.05, **p < 0.01, ****p < 0.0001.

The thickness of single-species biofilms significantly decreased when exposed to a final concentration of 50% CSE. The corrected *p*-values for treating *F. nucleatum*, *P. gingivalis*, and *S. oralis* biofilms were 0.0090, 0.0224, and < 0.0001, respectively. There was no significant decrease in the thickness of the multi-species biofilms, with an *p*-value of 0.1282 (Figure 8a). Confocal images are shown in Figure 8b.

**FIGURE 9:**
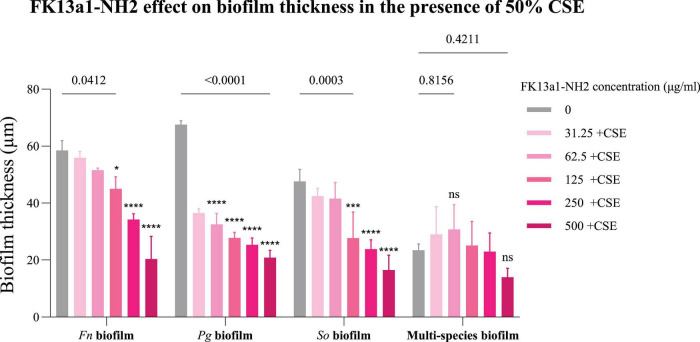
The efficacy of FK13a1-NH2 on biofilm thickness in the presence of 50% CSE using confocal imaging and live/dead staining. Statistical analysis was done by two-way ANOVA. The value points and error bars represent the mean and SD, respectively. The experiment involved three biological replicates. The comparison within each group is with the negative control (*Fn*, *Fusobacterium nucleatum*; *Pg*, *Porphyromonas gingivalis*; *So*, *Streptococcus oralis)*. ns, not significant, *p < 0.05, ***p < 0.001, ****p < 0.0001.

**FIGURE 10:**
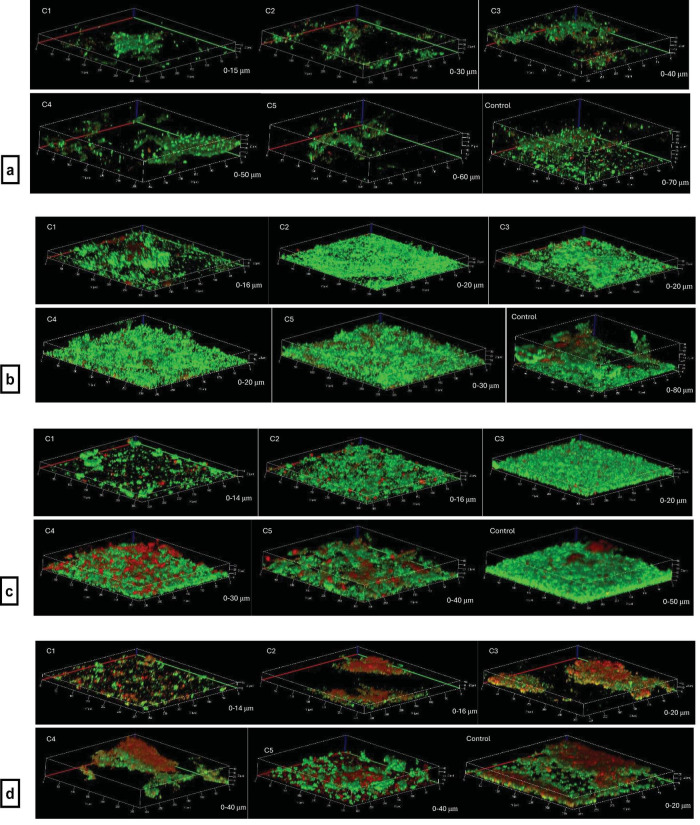
The confocal images of single- and multi-species biofilms with different concentrations (C1: 500 μg/ml, C2 250 μg/ml, C3 125 μg/ml, C4 62.5 μg/ml, C5 31.25 μg/ml) of FK13a1-NH2 plus 50% CSE. The most left bottom images in each set. **(a)** *F. nucleatum* biofilms. **(b)** *P. gingivalis* biofilms. **(c)** *S. oralis* biofilms. **(d)** multi-species biofilms. The magnification was 20x.

Confocal microscopy images of live/dead stained biofilms indicated that the thickness of *F. nucleatum* biofilms was significantly decreased by FK13a1-NH2 at 500, 250 (*p* < 0.0001), and 125 μg/ml (*p* = 0.0412), even in the presence of 50% CSE. *P. gingivalis* showed substantial decreases at concentrations of 62.5 μg/ml and higher (*p* < 0.0001). *S. oralis* biofilm thickness was significantly reduced at 500, 250 (*p* < 0.0001), and 125 μg/ml (*p* = 0.0003). However, no significant change was found in the thickness of the control biofilm versus the multi-species biofilms treated with the peptide in the presence of 50% CSE (Figure 9). The confocal images can be seen in Figure 10.

**FIGURE 11:**
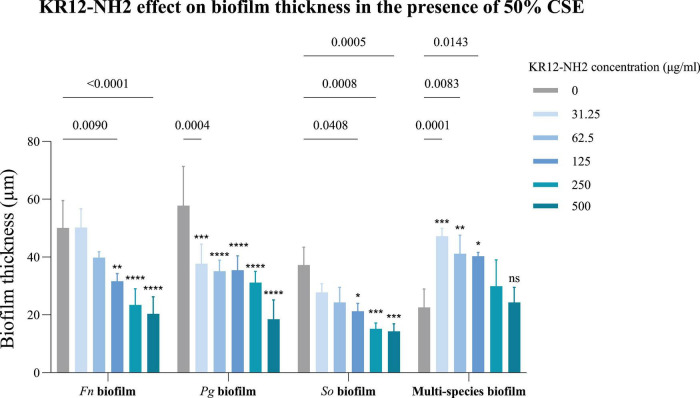
The efficacy of KR12-NH2 on biofilm thickness in the presence of 50% CSE using confocal imaging and live/dead staining. The data passed the Shapiro-Wilk normality test, and a two-way ANOVA was done. The value points and error bars represent the mean and SD, respectively. The experiment involved three biological replicates. The comparison within each group is with the negative control (*Fn*, *Fusobacterium nucleatum*; *Pg*, *Porphyromonas gingivalis*; *So*, *Streptococcus oralis*). ns, not significant, *p < 0.05, **p < 0.01, ***p < 0.001, ****p < 0.0001.

**FIGURE 12:**
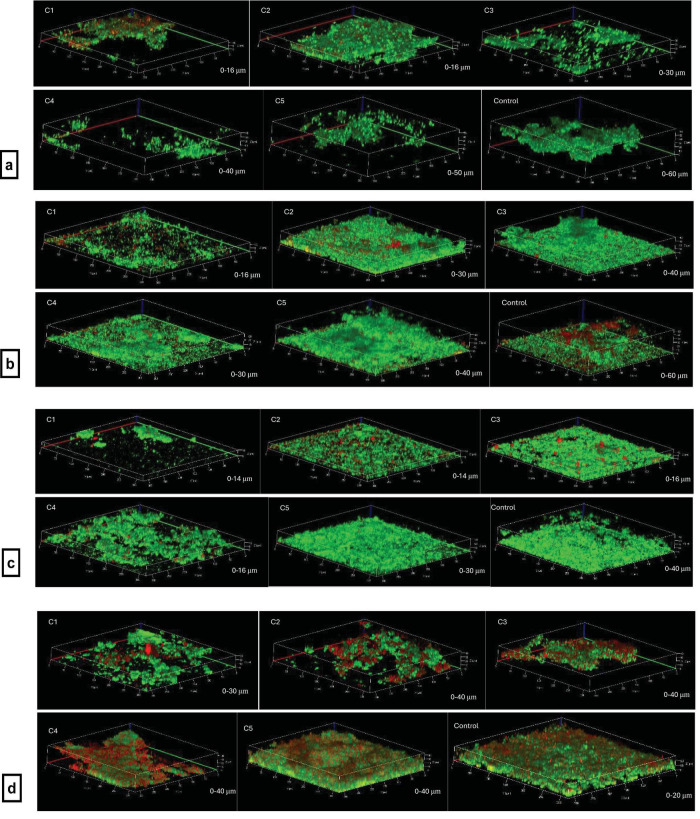
The confocal images of single- and multi-species biofilms with different concentrations (C1: 500 μg/ml, C2 250 μg/ml, C3 125 μg/ml, C4 62.5 μg/ml, C5 31.25 μg/ml) of KR12-NH2 plus 50% CSE. The most left bottom images in each set. **(a)** *F. nucleatum* biofilms. **(b)** *P. gingivalis* biofilms. **(c)** *S. oralis* biofilms. **(d)** multi-species biofilms. The magnification was 20x.

A significant decrease in the thickness of *F. nucleatum* biofilms was observed with 500 μg/ml and 250 μg/ml of KR12-NH2 in the presence of CSE (*p* < 0.0001), and with 125 μg/ml (*p* = 0.009). *P. gingivalis* biofilm thickness decreased significantly at 62.5 μg/ml and higher (*p* < 0.0001), as well as 31.25 μg/ml (*p* = 0.0004). A decrease in *S. oralis* biofilms was found at concentrations of 500 μg/ml (*p* = 0.0005), 250 μg/ml (*p* = 0.0008), and 125 μg/ml (*p* = 0.0408) (Figure 11). The confocal images can be seen in Figure 12.

The quantification of live and dead cells in biofilms was performed on raw Z-stack images using ImageJ and analysed via Dunnett’s multiple comparisons test against the negative control. The statistical significance of differences in live and dead cell populations (total and red values) between treated groups and the negative control varied by organism and combination can be seen in Table 3 and Supplementary Figures S2, S3.

**TABLE 3:** Quantitative assessment of total and dead cell counts in single- and multi-species biofilms following peptide treatment (with and without CSE) using confocal imaging.

Peptide	Organism	Viability metric	Without CSE (*p*-value)	With 50% CSE (*p*-value)
FK13a1-NH2	*F. nucleatum*	Total Cells	0.1622	0.1128
		Red (Dead)	0.2176	0.1288
	*P. gingivalis*	Total Cells	**0.0027**	**0.0009**
		Red (Dead)	**0.002**	**0.0006**
	*S. oralis*	Total Cells	0.2147	0.2108
		Red (Dead)	0.2789	0.197
	Multi-species	Total Cells	**0.0356**	**0.1897**
		Red (Dead)	**0.0365**	**0.2328**
KR12-NH2	*F. nucleatum*	Total Cells	0.6153	0.5862
		Red (Dead)	0.9817	0.9888
	*P. gingivalis*	Total Cells	0.0762	0.2549
		Red (Dead)	0.1323	0.2233
	*S. oralis*	Total Cells	0.5523	0.7597
		Red (Dead)	0.9564	0.9486
	Multi-species	Total Cells	0.2461	0.9955
		Red (Dead)	0.4197	0.9218

*P* < 0.05 are highlighted in bold.

## 4 Discussion

LL37 shows antibacterial, anti-osteoclast and anti-biofilm effects (Chinipardaz et al., 2022); the anti-biofilm effects of this peptide (Ridyard et al., 2023) are applied in several different ways; first, by reducing the adhesion of bacteria to the tooth surface, LL37 interferes with the first stages of biofilm formation; second, by disturbing the expression of genes related to quorum sensing, LL37 prevents signal communication between bacteria, which is essential for biofilm formation; third, by increasing the surface mobility of bacteria, this peptide decreases the thickness of biofilm; as a result, the identification and destruction of pathogenic bacteria are facilitated by the immune system (Vallabhan et al., 2016). Wuersching et al. suggested that the antimicrobial activity of LL37 is affected by the presence of oxygen in the environment; in this study, the inhibitory concentration of LL37 against biofilms was reported to be higher than that against planktonic bacteria, as the resistance of bacteria inside the biofilm is higher than that of unbound bacteria because the membranes of biofilm bacteria are less exposed to the surrounding environment containing antimicrobial peptides (Wuersching et al., 2021).

The results of the study were analysed and revealed that the two synthetic AMPs, FK13a1-NH2 and KR12-NH2, can effectively inhibit the growth of *F. nucleatum* and *P. gingivalis* in their planktonic phase. A clear difference in potency was established, with FK13a1-NH2 being significantly more potent than KR12-NH2; a lower MIC against *F. nucleatum* was recorded for FK13a1-NH2, and complete inhibition of *P. gingivalis* and *S. oralis* was achieved at the 500 μg/ml concentration. The high MIC values that were observed might be due to the high salt content in the BHI medium, which could interfere with how well these peptides worked. As Chu et al. pointed out, salt sensitivity is a major hurdle because it directly disrupts the way antimicrobial peptides interact with bacterial membranes, sometimes even rendering clinically active sequences useless. Their research showed that using a specific helix-hinge-helix structure can help solve this problem. Specifically, they discovered that increasing the hydrophobicity of the C-terminal end helped the peptide remain effective. This explains why their modified peptide could still kill bacteria at salt concentrations as high as 300 mM NaCl, demonstrating the importance of proper structural design in preventing salt from deactivating the peptide (Chu et al., 2020).

Both peptides effectively reduced the biofilm thickness of single-species biofilms, however, their effect on complex, multi-species biofilms were less pronounced. The variation that was observed is a well-established challenge. The efficacy of an AMP is not just about its interaction with the bacterial cell membrane, but rather a complex interplay between AMP efficacy, biofilm thickness, bacterial species and many other factors. The lower efficacy of AMPs against multi-species biofilms could be because of physiological barriers created by the Extracellular Polymeric Substance (EPS) matrix that acts as a physical and chemical shield that severely restricts the penetration of cationic AMPs that might trap by negatively charged components of the matrix, plus Efflux pumps can actively expel AMPs from the cell into the extracellular space (Singh et al., 2021); moreover, *S. oralis* was resistant to the peptides specially in the presence of CSE and it might contribute to the structural integrity of multi-species biofilms. This key finding underscores the need for higher-penetration delivery systems in clinical applications.

AMPs have represented a promising therapeutic alternative to traditional antibiotics for wound healing, demonstrating superior clinical efficacy in the management of severe periodontitis (Xiang et al., 2024). A review by Di Luca et al. highlighted studies showing that some AMPs were active against microbial biofilms, especially in the early phases of development. They offered potential for both prophylactic and therapeutic applications (Di Luca et al., 2014; Ridyard and Overhage, 2021). Similarly, in Li et al.’s study, the researchers observed that LL37 could inhibit the growth and structural integrity of *P. gingivalis* biofilms resulted in a noticeable reduction in biofilm thickness compared to untreated negative controls. This reduction occurs because the peptide disrupted the bacterial cell membranes and interfered with the initial adhesion of bacteria to the substrate, preventing the vertical accumulation and maturation of the biofilm layer (Li et al., 2025).

The mechanism of action of the AMPs, which involves disrupting bacterial membranes, can make them less susceptible to resistance (Ridyard and Overhage, 2021) because it is more difficult for bacteria to modify their whole membrane than bypassing the standard metabolic routes. Even if sensitivity decreases after many generations, the modular architecture of their sequences allows to be simply changed to counteract increasing resistance; For instance, by exchanging or reordering a particular residue found in the AlphaFold 3 models, such as arginines or tryptophans in FK13a1 and KR12. Given that these peptides exhibit high local confidence in their structural motifs, there is a clear blueprint for these future modifications.

It has been validated that smaller versions of LL37 can retain biological activity while reducing production costs and cytotoxicity (Chen et al., 2021); however, the safety assays must be performed before clinical trials for each derivative. Voronko et al. suggest that KR12-NH2’s higher amphipathic helicity than LL37 may have contributed to its increased antibacterial activity (Voronko et al., 2025). Furthermore, the resistance of the FK13 fragments to deactivation by bacterial components like LPS (Gupta et al., 2016) is particularly relevant to our findings regarding its efficacy. KR12 and FK13a1 are considered promising antimicrobial agents because they possess fewer protease cleavage sites than the 37-residue original (Voronko et al., 2025).

Smoking is known to promote an environment of microbial imbalance in the oral cavity, which persists even after periodontal therapy (Hanioka et al., 2019). While some studies show that quitting smoking can help restore a healthier microbiome (Brook and Gober, 2007), smoking actively reduces beneficial bacteria while increasing pathogenic ones (Kubota et al., 2011). In contrast, CSE increased the growth of *S. oralis* in our study. The results that CSE can inhibit certain bacteria but not others align with findings from Ertel et al. (1991), who showed that cigarette smoke has variable effects on different bacterial species. Similar differential effects were found on biofilm reduction, with single-species biofilms being more vulnerable to CSE than multi-species ones. This lends credence to the notion that bacteria in multi-species biofilms may work together to improve survival.

In contrast, Hutcherson and colleagues noted that tobacco components promote bacterial surface modifications that increase adherence to host tissues, effectively shifting bacteria from a free-floating state to a structured biofilm community (Hutcherson et al., 2015). By altering both the innate and adaptive immune systems, smoking was found to promote inflammatory signals that lead to an increase in autoimmune disorders, inflammatory diseases, and cancer. Consequently, while the primary objective is the cessation of smoking, particularly among patients requiring periodontal treatment, the habit has persisted despite these efforts. Therefore, the identification of novel antimicrobial agents capable of functioning within the harsh oral environment of smokers was deemed necessary (Dahdah et al., 2022). The presence of CSE was observed to lead to complex, species-dependent effects on peptide efficacy. Both KR12-NH2 and FK13a1-NH2 were effective against *P. gingivalis* and *F. nucleatum* in the presence of cigarette smoke. KR12-NH2 was not only insensitive to the presence of CSE in dealing with *P. gingivalis*, but the effect was actually greater when CSE was present. El-ezmerli et al. found that nicotine can enhance the growth of *S. mutans* biofilms (El-Ezmerli and Gregory, 2019) and increase its metabolic activity (Huang et al., 2012), reinforcing the necessity for strong anti-biofilm agents. Our results are also consistent with the findings of Li et al. (2014), who showed that nicotine can shift a healthy biofilm toward a disease-causing one by promoting the growth of *S. oralis*, which is a pioneer species in multi-species biofilms. The increased standard deviation observed in the presence of CSE in current study suggests that cigarette smoke might interfere with the consistent activity of the antimicrobial agent, a factor that warrants further investigation into peptide-smoke chemical interactions.

In the study conducted by Ingendoh-Tsakmakidis et al., human gingival fibroblasts were found to be extremely vulnerable to *S. oralis* biofilms, with significant harm and widespread cell loss being experienced by these cells which was related to a strong transcriptional stress response, and more than 300 genes linked to inflammation and apoptosis were activated (Ingendoh-Tsakmakidis et al., 2020). Therefore, a selective pressure could be created by *S. oralis* overgrowth under CSE stress, leading to the development of dysbiosis. This finding highlights that AMP treatment in smokers must be carefully optimised so that unintended shifts in the commensal flora are avoided.

This study provides a potential solution for a problem highlighted by Kzar et al. (2023) and Soldati et al. (2021), that smoking diminishes the body’s natural levels of protective AMPs like LL37. By introducing KR12-NH2 and FK13a1-NH2, effective LL37 derivatives, our study suggests a way to compensate for this deficit and inhibit *P. gingivalis* and probably *F. nucleatum* in smoking populations.

While a MEROPS analysis suggests that LL37 and its derivatives (FK13a1 and KR12) have theoretical cleavage sites for Arg-gingipain (RgpA), simple in vitro models usually overestimate how much degradation actually happens in a real-world setting. In actuality, LL37 is remarkably durable. This is most likely due to its tendency to form dimers and trimers in solution, a clustering propensity that keeps its concentration above the MIC even when proteases are present (Vallabhan et al., 2016).

Crucially, Gutner et al. identified what is essentially a salivary shield effect. They found that in whole saliva, LL37 is specifically protected from *P. gingivalis* proteases, even while other proteins in the same environment are easily degraded. This protective interaction doesn’t just stop the peptide from being destroyed; it helps trigger its antimicrobial activity (Gutner et al., 2009). Saliva may lessen the peptide’s initial effectiveness, but the trade-off of increased stability is more than acceptable. Because saliva shields LL37 from Arg-gingipain, the peptide remains active in the early stages of biofilm formation, which is essential for periodontal health. Since the peptides in this study were based on the active helical regions of LL37 and shared its strong cationic charge, it was expected that they behave in a similar way. They were designed to participate in these same protective interactions and oligomerization, making them much more stable in the complex oral environment than a basic protease map would suggest.

The various parameters of the human oral cavity cannot be perfectly replicated by the controlled *in vitro* environment in which our experiments were carried out. The long-term exposure in a smoker’s mouth could not be exactly reflected in the CSE concentrations. Future studies should investigate more physiologically relevant CSE concentrations and validate these results in more intricate *in vivo* models.

Determining the molecular pathways underlying the interaction between peptides and CSE should be investigated in future research. To understand how the mixture affects bacterial gene expression, contemporary methods like transcriptomics or proteomics are recommended. Kamysz et al. proposed that further research into chemical modifications such as lipidation could improve peptide potency and stability (Kamysz et al., 2020) which can improve the efficacy of these peptides for future studies. Ultimately, *in vivo* studies and clinical trials are the future logical steps to confirm the safety and efficacy of these peptides in treating periodontal disease in a real-world setting.

This study concluded that FK13a1-NH2 possessed a higher potency against all the tested bacteria than KR12-NH2 in the absence of CSE while KR12-NH2 was more effective against *P. gingivalis* in the presence of CSE, therefore, the combination of two could be viable alternative to traditional antibiotics in the treatment of periodontitis regardless of smoking status. However, more research is required to examine their effectiveness *in vivo.*

## Data Availability

The original contributions presented in the study are included in the article/Supplementary material, further inquiries can be directed to the corresponding author.
