# Supplementary material for: Differential antimicrobial activity of two LL37 derivatives against periodontal bacteria, including enhanced efficacy against P. gingivalis in the presence of cigarette smoke
Source: Front Microbiol. 2026 Apr 28;17:1804908. doi: 10.3389/fmicb.2026.1804908 (PMC13161148; doi:10.3389/fmicb.2026.1804908)
Supplement: Supplementary file 1 [file Table_1.docx]

Supplementary Material

# Supplementary Data

Supplementary Material should be uploaded separately on submission. Please include any supplementary data, figures and/or tables.

Supplementary material is not typeset so please ensure that all information is clearly presented, the appropriate caption is included in the file and not in the manuscript, and that the style conforms to the rest of the article.

# Supplementary Figures and Tables

For more information on Supplementary Material and for details on the different file types accepted, please see [here](https://www.frontiersin.org/guidelines/author-guidelines#supplementary-material).

## Supplementary Figures


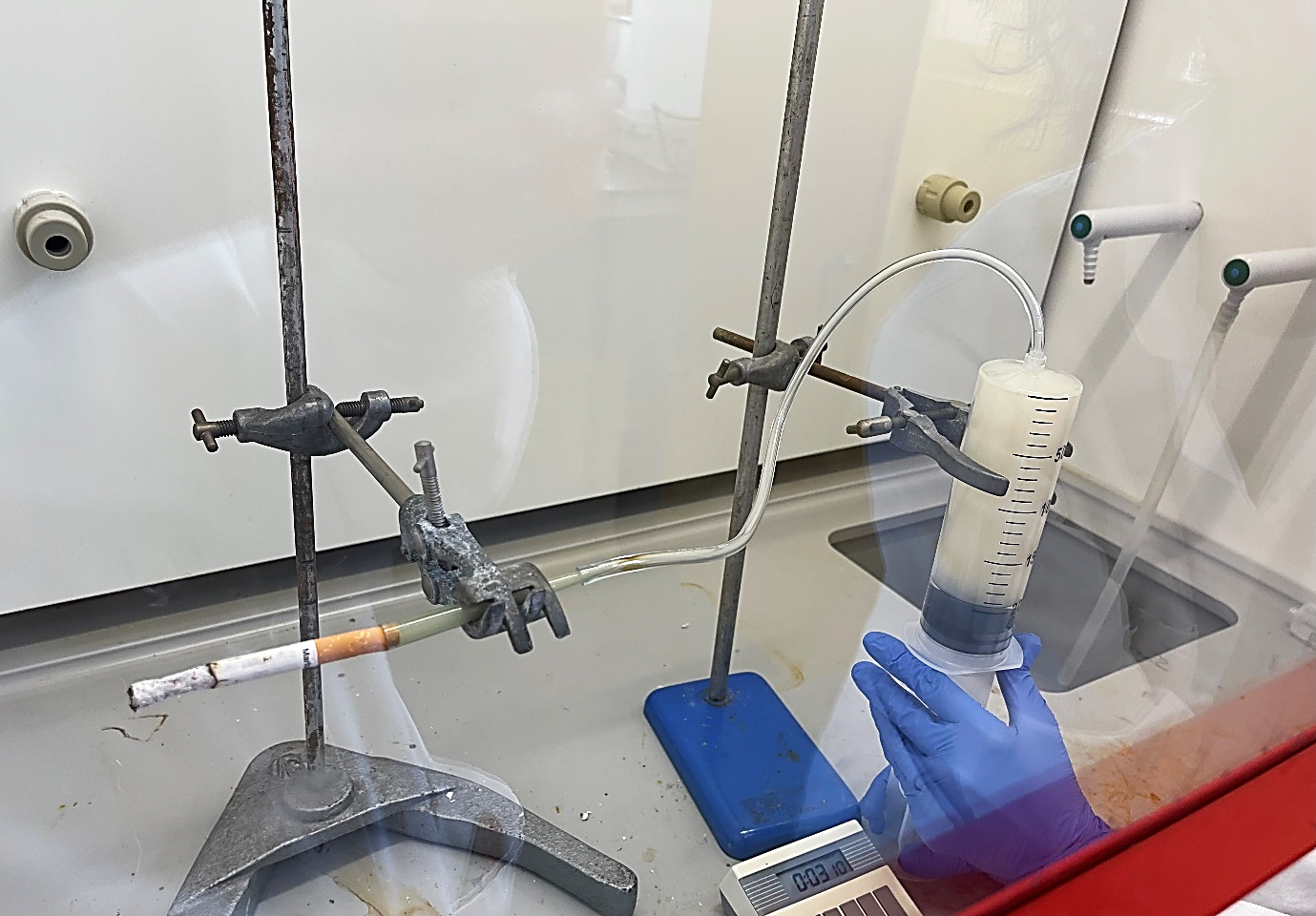


Figure S1. The tool for cigarette smoke extraction.

Figure S2. Confocal microscopy analysis of biofilms biomass. The effects of the treatments with different concentrations of FK13a1-NH2 in the presence and absence of 50% CSE were quantified in single- and multi-species biofilms. Live cells are represented by the green area, and dead cells are represented by the red area. Statistical comparisons were made using Dunnett's multiple comparisons test to evaluate the efficacy of the treatments against the negative controls. The value points and error bars are median and range, respectively. The experiments included three biological replicates in each experiment. a. *F. nucleatum* biofilms. b. *P. gingivalis* biofilms. c. *S. oralis* biofilms. d. multi-species biofilms.

Figure S3. Confocal Microscopy Analysis of Biofilm Treatments. The effects of the treatments with different concentrations of KR12-NH2 in the presence and absence of 50% CSE were quantified in various bacterial biofilms. Live cells are represented by the green area, and dead cells are represented by the red area. The data passed the normality test. The value points and error bars are median and range, respectively. Statistical comparisons were made using Dunnett's multiple comparisons test to evaluate the efficacy of the treatments against the negative controls. The experiments included three biological replicates in each experiment. a. *F. nucleatum* biofilms. b. *P. gingivalis* biofilms. c. *S. oralis* biofilms. d. multi-species biofilms.
